# Ghostwriting at Elite Academic Medical Centers in the United States

**DOI:** 10.1371/journal.pmed.1000230

**Published:** 2010-02-02

**Authors:** Jeffrey R. Lacasse, Jonathan Leo

**Affiliations:** 1 School of Social Work, College of Public Programs, Arizona State University, Phoenix, Arizona, United States of America; 2 Lincoln Memorial University - DeBusk College of Osteopathic Medicine, Harrogate, Tennessee, United States of America

## Abstract

Jeffrey Lacasse and Jonathan Leo assess ghostwriting policies at 50 academic medical centers in the United States and find that only 10 explicitly prohibit ghostwriting.

## Background

Medical ghostwriting, the practice of pharmaceutical companies secretly authoring journal articles published under the byline of academic researchers, is a troubling phenomenon because it is dangerous to public health [1]. For example, ghostwritten articles on rofecoxib [2] probably contributed to “…lasting injury and even deaths as a result of prescribers and patients being misinformed about risks” [3]. Study 329, a randomized controlled trial of paroxetine in adolescents, was ghostwritten [4]–[7] to claim that paroxetine is “generally well tolerated and effective for major depression in adolescents” [8], although data made available through legal proceedings show that “Study 329 was negative for efficacy on all 8 protocol specified outcomes and positive for harm” [9]. Even beyond frank misrepresentation of data, commercially driven ghostwritten articles shape the medical literature in subtler but important ways, affecting how health conditions and treatments are perceived by clinicians. The ability of industry to exercise clandestine influence over the peer-reviewed medical literature is thus a serious threat to public health [1],[10].

In 2009, the Institute of Medicine recommended that US-based academic medical centers enact policies that prohibit ghostwriting by their faculties [11]. However, to date, there has been no systematic assessment of ghostwriting policies at academic medical centers. Since US-based academic medical centers generate biomedical research for a worldwide audience, we chose to conduct the first such investigation on elite US-based academic medical centers. Our methods are shown in Box 1. We sought to describe the current policy situation at US-based academic centers and then to propose an ideal ghostwriting policy.

**Box 1. Methods**

At the beginning of the 2009–2010 academic year, we evaluated the policies of the top-50 academic medical centers by research ranking according to the 2009 US News and World Report [29]. To avoid response bias, and given that faculty policies are commonly published on the World Wide Web, we searched for publicly available policy documents. We used a standardized search protocol in the Google search engine and key phrases used in policies regulating authorship, ghostwriting, and conflicts of interest. When we were unable to locate a published authorship policy, we contacted a reference librarian at the institution to verify that no policy was available. We also searched each Web site to see if any conflict-of-interest policies or faculty manuals were available on-line. We retrieved only policies that were publicly available and applicable to the entire academic medical center. Our retrieval method removed social desirability bias as a possible confounder but was time-intensive, leading us to examine only the top-50 schools, a trade-off we found reasonable given the influential nature of elite US-based medical schools in the worldwide biomedical research community and the exploratory nature of this research.

One rater (JRL) extracted data from the policies. If an academic medical center explicitly prohibited ghostwriting, this was coded as such, and the policy was transcribed. If ghostwriting was not mentioned, but there was an authorship policy, the policy was coded on whether it mandated (1) a substantive contribution to qualify for authorship and (2) that all individuals who make substantial contributions to the manuscript be listed as authors. Inclusion of both (1) and (2) was coded as prohibiting ghostwriting in practice.

To ensure reliability, two sets of data were blindly recoded by the second author (JL). First, a 50% random sample of those medical centers coded as lacking any authorship policies was recoded. There was disagreement on the existence of an authorship policy at one institution, which was resolved through discussion. Second, a 50% random sample of institutions with authorship policies were recoded on the two primary variables of interest, with perfect agreement between the two raters. All data are available as an Excel spreadsheet file, which includes hyperlinks to each institution's policies (Dataset S1), or as a PDF file (Dataset S2).

## Findings of Our Survey

Of the 50 academic medical centers that we examined (Box 1), ten (20%) explicitly prohibit ghostwriting. Of these ten, seven (14%) include some definition of ghostwriting in their policy, while three (6%) prohibit ghostwriting without defining the term. Many schools have an authorship policy that does not clearly ban all aspects of ghostwriting (*n* = 13, 26%); the most common reason is a failure to require that all qualified authors be listed. Three academic medical centers (6%) have stringent authorship policies that prohibit it in practice (by requiring both a substantive contribution to qualify for authorship and that all who qualify for authorship be listed) but do not mention ghostwriting by name (Table 1).

**Table 1:** Published policies of academic medical centers meeting specific criteria (*n* = 50).

Criteria	*n*	%
Some faculty policies available on-line	45	90
Ghostwriting explicitly banned	10	20
Ghostwriting explicitly banned and defined in some way	7	14
Authorship policy that does not mention ghostwriting	13	26
Authorship policy requires substantial contribution for authorship	9	18
Authorship policy requires all those that qualify as authors must be listed as such	3	6
Policy that bans ghostwriting in practice	13	26
No published policy on either authorship or ghostwriting	26	52

By combining the ten schools that explicitly ban ghostwriting with the three schools that have authorship policies banning it in practice, we find that 13 of the top-50 academic medical centers (26%) have policies in place prohibiting medical ghostwriting. Six of the top-ten schools ban ghostwriting in practice, and all top-ten academic medical centers have published authorship policies. Although most schools (*n* = 45, 90%) had some policy documents posted online, the majority of academic medical centers (*n* = 26, 52%) had no published policies at all on either ghostwriting or authorship. The Web sites of two schools stated that they did have such policies, but the policies were not currently available online.

## Implications of These Findings

A minority of top-50 US-based academic medical centers (*n* = 13, 26%) publicly prohibit their faculty from participating in ghostwriting. It is ironic that ghostwriting, a major threat to public health, is generally not prohibited within institutions that exist to train physicians and improve the public health. In this way, academic medical centers enable the pharmaceutical industry to covertly shape the medical literature in favor of commercial interests. When a pharmaceutical salesperson hands a clinician an article reprint, the name of the institution on the front page of the reprint serves as a stamp of approval. The article is not viewed as an advertisement, but as scientific research; the reprint is an effective marketing tool because peer-reviewed journal articles generated in academia are perceived to be the result of unbiased scientific inquiry. Deception regarding authorship prevents a discriminating audience from properly assessing the impact of bias in the published article [10]. Importantly, this deception is impossible without the cooperation of faculty employed by academic medical centers.

The practice of ghostwriting explicitly violates the usual norms of academia. We are not aware of any other academic fields where it is acceptable for professors to allow themselves to be listed as authors on research papers they did not write, or to purposefully conceal the contributions of industry coauthors in order to mislead readers. A recent *New York Times* article characterizes medical ghostwriting as “an academic crime akin to plagiarism” [12]. Anecdotally, we find many of our academic colleagues are stunned to hear about ghostwriting in medical schools, and some of our graduate students express dismay. (They have to write their own papers, and face disciplinary action and even expulsion if they submit term papers they did not write). In contrast, academic medical centers in the US and Europe employ professors who are publicly known to have participated in ghostwriting (e.g., [4]–[6],[13]). The culture of biomedical research apparently condones or at best takes a neutral position when it comes to ghostwriting. This suggests that ghostwriting will continue to be a problem until policy solutions are implemented. While our survey examined only published policies, the dearth of such policies is cause for concern.

Perhaps ghostwriting policies should be examined in the context of existing policies meant to regulate ethical research behavior. It is possible that some academic medical centers already prohibit ghostwriting under other rules of research integrity. For instance, ghostwriting may be characterized as a form of plagiarism [14], and to our knowledge, all academic institutions consider plagiarism to be a form of academic misconduct. Some academics have listed ghostwritten publications on their curricula vitae, meaning that they were considered for promotion and/or grants on the basis of fraudulent authorship, which would seem to be grounds for disciplinary action. It has been reported that academics receive payments from industry for participation in ghostwriting, and many institutions have rules requiring faculty to report outside income. Failure to report such income truthfully may violate existing policies. In theory, an administrator could penalize a violation of such policies by a faculty member who has participated in ghostwriting. If any of this has ever occurred, it is not publicly known.

A policy is only as useful as it is enforceable. A policy prohibiting ghostwriting that cannot be effectively enforced is unlikely to change practice. It is worth considering, then, whether existing policies of academic medical centers regulating authorship and ghostwriting clearly define “ghostwriting”? Is a policy useful if it forbids ghostwriting but never defines the term? Can we envision an academic being sanctioned for violating a policy that does not define its critical terms? Or does this lack of clarity provide “wiggle room” to evade sanctions? Our review of existing ghostwriting policies (see Datasets S1 and S2) indicates that the clarity of many policies could be improved substantially. For instance, the *New York Times* reported that Duke University has a policy which bans ghostwriting [15]. On closer examination, what Duke's policy prohibits is courtesy authorship—but it does not require that all contributors who qualify as authors be listed as such. The policy requires that a substantial contribution be made to qualify for authorship, but does not prohibit the concealment of corporate writers in the preparation of the manuscript. A professor could follow this policy to the letter and still participate in something most people would call “ghostwriting” [16] by failing to list a corporate coauthor in the author byline. Other existing ghostwriting policies have similar deficiencies and ambiguities.

## An Unambiguous Policy Proposal

Ghostwriting was once the “dirty little secret” of the medical literature [3], but this no longer is the case. Pharmaceutical companies have used ghostwriting to market sertraline [17], olanzapine [18], gabapentin [19], estrogen replacement therapy [20], rofecoxib [2], paroxetine [4],[21], methylphenidate [22], milnaciprin [23], venlafaxine [24], and dexfenfluramine [25]. Ghostwriting is now known to be a major industry [26].

In the near future, we expect administrators of academic medical centers to enact policies that regulate medical ghostwriting. Such policies must be operationalized specifically enough to actually change practice. A problematic policy may be worse than no policy at all, as it may give the misleading impression that the ghostwriting problem has been solved. Therefore, we make the following policy proposal to academic medical centers worldwide.

### The Proposal

First, deans of academic medical centers should immediately inform their faculties that a ban on medical ghostwriting will be enacted shortly. Following the suggestion by Barton Moffatt and Carl Elliot [1], the remaining months in the 2009–2010 academic year should be a period of amnesty. Faculty who have participated in ghostwriting will be allowed to come forward and describe their involvement. Known ghostwritten papers should be reevaluated by the academic medical community and considered for retraction.

Next, a policy that clearly defines participation in ghostwriting as a form of academic misconduct should be implemented at the beginning of the 2010–2011 academic year. By modifying several existing authorship policies to close any loopholes and be as specific as possible, we suggest the following wording:

“All listed authors on a publication must meet the authorship criteria set by the International Committee of Medical Journal Editors. Making minor revisions to a manuscript does not qualify as authorship. Participating in the creation of ghost-authored manuscripts is not permitted. A ghost author is defined as someone who makes substantial contributions to writing a publication but is not listed as an author. All individuals who have made a substantial contribution to the manuscript must be listed as authors. Accurately reporting authorship is essential for maintaining research integrity, and violating any of these rules is considered research misconduct akin to plagiarism or falsification of data.”

### Implementation and Enforcement

Government funding agencies can play a primary role in encouraging the adoption of this policy. Francis Collins, Director of the US National Institutes of Health (NIH), recently remarked that “I was shocked by that revelation—that people would allow their names to be used on articles they did not write, that were written for them, particularly by companies that have something to gain by the way the data is presented…if we want to have the integrity of science preserved—that's not the way to do it” [27]. We agree, and suggest that, to encourage the adoption of this policy, NIH and similar funding agencies should refuse to disperse any public research funds to institutions that do not adopt a policy which bans ghostwriting, as we have suggested above. Academic medical centers are funded with public monies because they ostensibly serve the public good. Since ghostwriting harms public health and serves commercial rather than public interests, governments should not support institutions that permit ghostwriting.

**Figure 1:**
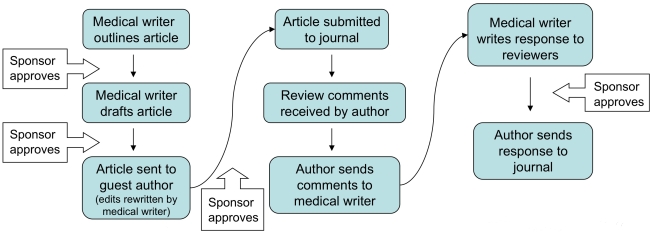
Acknowledging ghostwriters does not accurately reflect their authorship role. Modified from [14]. Used under a Creative Commons license which permits the modification and re-use of intellectual content as long as it is properly acknowledged.

At the institutional level, vigorous enforcement efforts should accompany the implementation of such policies. Administrators should carefully monitor the medical literature for clues of ghostwriting, such as an acknowledgment of a medical writer's assistance in a peer-reviewed journal article. When a medical writer is thanked, this will be taken to mean that they do not qualify for authorship, much in the way that a copyeditor does not receive a byline credit. At present, such acknowledgments are suspected to mean that the medical writer actually ghostwrote the paper (Figure 1) [28], but the implementation of a stringent ghostwriting policy will require strict accuracy on this issue. When there is doubt, aggressive investigative action should be taken. The empirical findings of medical literature are unlikely to change, but reports of authorship would thus be honest and transparent.

When it comes to light that an academic has violated this policy, rapid disciplinary action should result. Sanctions should be equivalent to those used in cases of plagiarism or falsification of data. When a behavior poses a significant public health risk, most governments punish such behavior vigorously. For instance, most governments heavily penalize people who drive an automobile while intoxicated; the goal is to protect the public by deterring the behavior. Similarly, it is hard to envision a policy that protects the public from ghostwriting without punishing the behavior.

Ultimately, this policy requires only that academic medical centers follow the norms of science, as exemplified by other departments of the university. Honest and transparent reporting of authorship has always been an essential element of scientific communication. We can think of no ethical or scientific reason why this proposal should not be adopted by every academic medical center.

## Conclusion

Medical ghostwriting is a threat to public health which currently takes place only due to the cooperation of researchers employed at academic medical centers. Although there is growing awareness of the danger posed by medical ghostwriting, we find that few academic medical centers have public policies which prohibit this behavior, and many of the existing policies are ambiguous or ill-defined. We have proposed an unambiguous policy which defines participating in medical ghostwriting as academic misconduct akin to plagiarism or falsifying data. By adopting and enforcing this policy, academic medical centers would adhere to the norms of science followed across the rest of the University, and would no longer facilitate clandestine industry influence over the peer-reviewed scientific literature. By prohibiting medical ghostwriting, academic medical centers have a rare opportunity- to significantly reduce a major threat to public health with the stroke of a pen.

## Supporting Information

Dataset S1 Ghostwriting policies by academic medical center - Raw data file in Microsoft Excel format.
(0.07 MB XLS)

Dataset S2 Ghostwriting policies by academic medical center - Raw data file in PDF format.
(0.09 MB PDF)
