# Supplementary material for: Ghostwriting at Elite Academic Medical Centers in the United States
Source: PLoS Med. 2010 Feb 2;7(2):e1000230. doi: 10.1371/journal.pmed.1000230 (PMC2814828; doi:10.1371/journal.pmed.1000230)
Supplement: Dataset S2 — Ghostwriting policies by academic medical center - Raw data file in PDF format. (0.09 MB PDF) [file pmed.1000230.s002.pdf]

U.S. News and World Report Medical School Research Ranking (2009)	School Name	Public or Private?	Web Link to Authorship Policy	Is ghostwriting explicitly banned?
1	Harvard Medical School	Private	Policy	No
2	Johns Hopkins University	Private	Policy	**Yes**
3	University of Pennsylvania	Private	Policy	No
3	Washington University in St. Louis	Private	Policy	**Yes**
5	University of California- San Francisco	Public	Policy	No

6	Duke University	Private	Policy	No
6	Stanford University	Private	Policy	**Yes**
6	University of Washington	Public	Policy	**Yes**
6	Yale University	Private	Policy	No
10	Columbia University	Private	Policy	**Yes**

11	University of California- Los Angeles	Public	None	No
11	University of Michigan- Ann Arbor	Public	None	No
13	University of Chicago	Private	None	No
13	University of Pittsburg	Public	Policy	No
15	University of California- San Diego	Public	None	No
15	Vanderbilt University	Private	None	No
17	Baylor College of Medicine	Private	None	No
18	Cornell University	Private	Policy	No
19	Northwestern University	Private	Policy	**Yes**
20	University of North Carolina- Chapel Hill	Public	None	No
20	University of Texas Southwestern Medical Center-	Public	None	No

22	Emory University	Private	Policy	No
22	Mount Sinai School of Medicine	Private	Policy	No
24	University of Virginia	Public	Policy	No
25	Case Western Reserve University	Private	Policy	No
25	Mayo Medical School	Private	None	No
27	Ohio State University	Public	None	No
27	University of Wisconsin- Madison	Public	None	No
29	University of Alabama-Birmingham	Public	None	No

29	University of Colorado- Denver	Public	Policy	**Yes**
31	New York University	Public	None	No
31	University of Iowa	Public	Policy	**Yes**
31	University of Rochester	Private	None	No
34	Brown University	Private	None	No
35	Boston University	Private	Policy	No
35	Dartmouth Medical School	Private	None	No
35	Oregon Health and Science University	Public	Link only works from within OHSU	No
35	University of Minnesota	Public	None	No
39	Georgetown University	Private	None	No
39	University of Southern California	Private	None	No

41	University of Cincinnati	Public	None	No
41	Yeshiva University	Private	None	No
43	University of Maryland	Public	Policy	**Yes**
43	Wake Forest University	Private	Policy	No
45	Indiana University	Public	None	No
45	Tufts University	Private	Policy	Yes
45	University of California- Irvine	Public	None	No

48	University of California- Davis	Public	None	No
48	University of Florida	Public	None	No
48	University of Massachusetts- Worcester	Public	None	No

Supporting citation	If ghostwriting is not explicitly banned, does the school have an authorship policy that would prohibit ghostwriting by both (1) requiring substantive contribution to qualify for authorship, and (2) requiring that all authors who made substantial contributions be listed?	Is a substantive contribution required for authorship?
	**Yes**	Yes
""Ghost-writing," a practice whereby a commercial entity or its contractor writes an article or manuscript and a scientist is listed as an author, is not permissible. Making minor revisions to an article or manuscript that is ghost-written does not justify authorship."		
	**No; lacks (2)**	Yes
"Ghost or guest authorship is unacceptable and a violation of this policy. A ghost or guest author is defined as someone who made substantial contributions to writing a publication, gives credit for the work to another individual, and whose contributions are not mentioned in the publication."		
	**No**	Yes

	**No; lacks (2)**	Yes
"Individuals are prohibited from ghost-writing; in other words, individuals may not publish articles under their own names that are written in whole or material part by industry employees."		
"UW SOM faculty, trainees, students, and staff may not serve as authors on any publications in which ghost authorship has occurred."		
	**No; lacks (2)**	Yes
"…Faculty, trainees and students are prohibited from authoring or co-authoring articles written by employees of commercial entities, where their name or Columbia title is used without substantive contribution. If commercial employees are co-authors, they should be acknowledged as such. Any such articles or other materials written in conjuction with commercial entities must include full disclosure of the role of each author, as well as other contributions or participation by such commercial entities. P & S Faculty authors who collaborate with commercial entities must retain mantain editorial independence at all times."		

	**Yes**	Yes
	**No**	No
"Faculty, students, and trainees are prohibited from allowing their professional presentations of any kind, oral or written, to be ghostwritten by any party, industry or otherwise."		

	**No, lacks (2)**	Yes
	**No, lacks (2)**	Yes
	**No, lacks (2)**	Yes
	**No, lacks (2)**	Yes

"HSC students, residents and faculty may not be listed as authors or co-authors on papers, monographs or other publications that are ghost-written by industry representatives."		
"Faculty, staff, and trainees may not participate in ghost authorship as either the named or unnamed author. Ghost authorship exists when someone has made substantial contributions to writing a manuscript and this role is not mentioned in the manuscript itself."		
	**No, lacks (2)**	Yes
	**Yes**	Yes

"School faculty and students may not allow their professional scholarly papers or presentations of any kind, oral or written, to be ghostwritten by any person from industry, a medical education company, or another source. ""Ghostwriting"" refers to claiming authorship of a paper or presentation that was written or prepared by another person."		
	**No, lacks (2)**	Yes
"TUSM Faculty participation in ghostwriting of pharmaceutical research, in which authors represent industry research as their own, is prohibited."		

Supporting citation	Is it required that all authors who make substantive contributions to the manuscript must be listed as co-authors?
"Everyone who is listed as an author should have made a substantial, direct, intellectual contribution to the work. For example (in the case of a research report) they should have contributed to the conception, design, analysis and/or interpretation of data."	Yes
"Conditions 1,2, and 3 [ICMJE] must all be met in assignment of authorship"	Yes
"Authorship must reflect substantial scientific involvement in the research being reported."	No

"Authorship should be restricted to those individuals who have met EACH of the following three criteria: 1) made a significant contribution to the conception and design of the project, or the analysis and interpretation of the data, or other substantial scholarly effort; 2) participated in drafting, reviewing and/or revising the work, and 3) approved the final version for publication."	No
"Authorship of a scientific paper should be limited to those individuals who have contributed in a meaningful way to its intellectual content. Each author must have participated sufficiently in the work to take public responsibility for its content."	No

"Authorship credit should be based only on a) substantial contributions to conception and design, or acquisition of data, or analysis and interpretation of data and b) final approval of the version to be published. Conditions a and b must be met."	Yes
"In general, all persons who have made significant intellectual or functional contributions to a study should be included among the co-authors. However, no universally accepted rules exist regarding authorship and order of authorship."	No

"Authorship should be granted to, and only to, those persons who have made appropriate contributions to the conceptualization, design, execution, or interpretation of the work reported."	No
"All persons designated as authors should qualify for authorship. The order of authorship should be a joint decision of the coauthors. Each author should have participated sufficiently in the work to take public responsibility for the content. Authorship credit should be based only on substantial contributions to (a) conception and design, or analysis and interpretation of data; and to (b) drafting the article or revising it critically for important intellectual content; and on (c) final approval of the version to be published. Conditions (a), (b), and (c) must all be met."	No
"To be listed as an author of an original scientific article, an investigator must have: (a) made substantial contributions to the conception, design, or data acquisition, analysis, or interpretation of the project; (b) drafted or critically revised the resulting manuscript; and (c) given approval of the manuscript to be submitted for publication."	No
"Authorship is attributed to persons responsible for the intellectual content of the publication. Only those who have contributed substantially to the conception, execution, or interpretation of the work such that they are willing and able to take public responsibility for the publication should be included as authors."	No

"…2) Each author must have made a substantial contribution to the actual drafting or writing of the manuscript; 3) Each author must have signed off on the final manuscript."	No
"A substantial contribution to one or more of these activities [e.g., write a major portion of the paper; analyze data] is generally considered sufficient for authorship."	Yes

"Authorship should be given generously, but only to those who have contributed significantly to the research, are prepared to stand behind their findings, and have reviewed the entire manuscript. All authors of a scholarly publication should meet the following four criteria: Participate substantially in conception, design, and execution of the study, or in the analysis and interpretation of data;Participate substantially in the drafting of the manuscript or in the substantive editing of the manuscript; Give final approval of the version of the manuscript to be published; and Be able to explain and defend in public or scholarly settings that portion of the study for which he or she was directly responsible, including potential conflicts of interest."	No

Supporting citation	Is courtesy or honorary authorship explicitly prohibited?	Supporting citation
"Everyone who has made substantial intellectual contributions to the work should be an author."	Yes	"Honorary or guest authorship is not acceptable."
"Authorship credit should be based only on substantial contributions to each of the following areas:…"	No	
	No	

	Yes	"Honorary or courtesy authorships are inconsistent with the principles of this policy, and, as such, are unacceptable."
	Yes	"Individuals do not satisfy these criteria for responsible authorship merely because they have made possible the conduct of the research and/or the preparation of the manuscript. Under no circumstance should faculty members add as co-authors highly respected individuals merely as an attempt to increase the likelihood of publication. Thus, heading a laboratory, research program, section, or department where the research takes place does not, by itself, warrant co-authorship of a scientific paper. Nor should "gift" co-authorship be conferred..."

"All persons designated as authors should qualify for authorship, and all those who qualify should be listed."	No	
"In general, all persons who have made significant intellectual or functional contributions to a study should be included among the co-authors. However, no universally accepted rules exist regarding authorship and order of authorship."	No	""Honorary" or "courtesy" authorship is generally viewed as undesireable, since it distorts issues of responsibility and credit. In addition it should be emphasized that "honorary" or "courtesy" authorship carries with it all of the responsibilities and potential liabilities of authorship. Nevertheless, as a consequence of particular conditions, specific agreements between or among faculty with regard to authorship practices may be entered into formally or informally.

	No	
	No	
"All individuals who meet these three criteria should be listed as authors; however, any individual may refuse to be included as a co-author."	Yes	"Honorary or courtesy authors should not be included."
	No	"Honorary authorship, that is, listing someone as a coauthor in the absence of substantial intellectual contribution, is discouraged."

	No	
"While technical contributions may not necessarily require acknowledgment, a substantial intellectual contribution must be recognized by inclusion of the individual's name as an author"	No	

	Yes	"The practice of permitting honorary authorship is unacceptable and should be actively discouraged."

Notes	Web Link to Faculty Handbook	Web Link to COI Guidelines

		Policy
		Policy
		Policy
	Policy	Policy

		Policy
		Policy
		Policy
		Policy

		Policy
	Policy	
	Policy	
There is an authorship policy found in the Office of Sponsored Projects, but the criteria for authorship are presented as "guidelines" and are said to vary by discpline.		
		Policy
	Policy	
	Policy (Dead Link)	

Conflict of Interest PolIcy States: "Publications which are supported by and/or include contributions from industry author(s) shall strictly comply with "Authorship Guidelines- Faculty University of Cincinnati College of Medicine (May 13, 2004) as amended from time to time." (This document not publicly available).		Policy
No current policy posted, but previously had an authorship policy posted		Policy
		Policy
		Policy

		Policy
		Policy
		Policy
